# The OrBiD (Oral Health, Bite Force and Dementia) Pilot Study: A Study Protocol for New Approaches to Masticatory Muscle Training and Efficient Recruitment for Longitudinal Studies in People with Dementia

**DOI:** 10.3390/ijerph19063700

**Published:** 2022-03-20

**Authors:** Julia Jockusch, Daniel Wiedemeier, Ina Nitschke

**Affiliations:** 1 Department of Prosthodontics and Materials Science, Gerodontology Section, University of Leipzig, Liebigstraße 12, 04103 Leipzig, Germany; ina.nitschke@medizin.uni-leipzig.de or; 2 University Research Priority Program “Dynamics of Healthy Aging”, University of Zurich, Andreasstrasse 15/Box 2, CH-8050 Zurich, Switzerland; 3 Center of Dental Medicine, University of Zurich, Plattenstrasse 11, CH-8032 Zurich, Switzerland; daniel.wiedemeier@zzm.uzh.ch; 4 Clinic of General, Special Care and Geriatric Dentistry, Center of Dental Medicine, University of Zurich, Plattenstrasse 11, CH-8032 Zurich, Switzerland

**Keywords:** dementia, recruitment process, study protocol, oral health, chewing efficiency, bite force, oral hygiene, Mini–Mental State Examination, muscle training, physiotherapy

## Abstract

Research with people with dementia is a great challenge in terms of recruitment, study participation and adherence to interventions resulting in less research activity and higher financial, organizational and personnel efforts. As dementia progresses, there is a deterioration in general and oral health and chewing function. Oral treatment options often focus on healthy patients. Interventions for people with dementia are needed. The aims of the paper were to describe the study protocol of the OrBiD (Oral Health, Bite Force and Dementia) pilot study as well as the description of two new methodological approaches. These are (A) an efficient recruitment process of people with dementia, simultaneous double study participation and (B) a novel approach to train the masticatory muscles by using physiotherapy. A novel methodology for the recruitment process (A) and, in particular, for the assignment of subjects to the experimental and control groups was developed and successfully tested. Additionally, a physiotherapy program (B) to train strength and coordination of the masticatory muscles was newly developed with the challenge to ensure that this training could also be carried out with people with cognitive impairments and dementia, if necessary, in cooperation with their relatives or caregivers. This was also successfully implemented. Recommendations for a feasibility assessment of a study involving people with dementia were made considering the organizational effort, the required personnel, structural and financial resources, the required number of subjects and the type of study design. When planning crossed studies, it must be ensured that the content, the interventions or their possible results of the study arms do not influence each other. The overall aim of this paper is to demonstrate the sustainable and efficient feasibility of studies with people with dementia.

## 1. Introduction

Dementia is one of the greatest challenges for society in terms of the increasing number of people suffering from it [1]. As dementia progresses, there is a deterioration in the general health of those affected, which in turn affects their daily lives and their families.

Due to demographic trends and the increasing number of people who retain their teeth into old age, dentists also have to continue to focus on the care of the old and eldest. The challenge for this patient group lies in recognizing the heterogeneity of the functional capacity of each individual patient, which is based on multimorbidity and polypharmacy, social isolation and financial resources, and assessing this heterogeneity correctly for the benefit of the patient. The challenge can only be met if both profound dental knowledge of gerodontology and interdisciplinary knowledge (e.g., geriatrics, geronto-psychiatry, ethics, nursing sciences, care research, etc.) are present [2,3,4].

Recruiting people with dementia for research is always a major challenge for the study staff. It takes a long time, and intensive information has to be provided not only to the subjects but also to their relatives and often to the management of the care facility. This consumes a lot of research resources, which makes studies with people with dementia more expensive, more strenuous, and thus comparatively rare.

There is little research on whether there is a relationship between the decline in cognitive performance and the deterioration in oral health or nutrition as a result of a reduced chewing function.

This paper provides a complex study protocol for the OrBiD (Oral health, Bite force and Dementia) pilot study. This study is designed to evaluate people with and without dementia regarding their oral health and chewing function (Supplementary Material File S1, [5,6,7,8,9,10,11,12,13,14,15,16,17,18,19,20,21]) both cross-sectionally and longitudinally in a pilot randomized clinical trial with interventions.

The aim of this paper was to describe the study design and applied methods of the OrBiD pilot study, including a profound look at the methodological peculiarities of this study. These are (A) an efficient recruitment process of people with dementia, simultaneous double study participation, and (B) a novel approach to train the masticatory muscles by using physiotherapy.

The overall aim of this paper is to demonstrate the efficient and thus resource-saving feasibility of studies with people with dementia at the example of the OrBiD pilot study.

## 2. Materials and Methods: Description of the OrBiD Pilot Study

### 2.1. Study Design

OrBiD (Oral Health, Bite Force and Dementia) (ClinicalTrials.gov NCT03775772) is a clinical, interventional pilot study with randomization and stratification.

The study consists of two study parts (A and B). Part A investigates oral health in people with and without dementia; Part B investigates bite force and chewing efficiency as a function of the degree of dementia. Both study parts differ in terms of their set-up and study design but use the same cohorts of study subjects. (Table 1) Both parts A and B are cross-sectional designs with inter-cohort analysis at the evaluation time-point T0 (Baseline). With regard to the other evaluation time-points (study part A—oral health: follow-up after 12 months (Final); study part B—chewing function: follow-up three (T1) and six months (T2) after the start of the intervention, and follow-up after 12 months (Final)), both study parts are prospective, clinical longitudinal designs with interventions (intra-cohort analysis) (Table 1; Supplementary Material File S2 [15,19,22,23,24,25,26,27,28,29,30,31,32,33,34,35,36,37]).

A total of 120 subjects were included in the study. Stratified randomization was used as follows: Since dementia was expected to have a major impact, the subjects were assigned to one of five MMSE groups (24 subjects each) after the first examination based on the evaluation of the Mini Mental State Examination (MMSE). These MMSE groups are 1—no dementia (MMSE 28–30), 2—mild cognitive impairment (MMSE 25–27), 3—mild dementia (MMSE 18–24), 4—moderate dementia (MMSE 10–17) and 5—severe dementia (MMSE ≤ 9). Each MMSE group was divided into an experimental group (*n* = 12) and control group (*n* = 12) for each study part. Within the MMSE groups, further allocation to the experimental group was carried out as follows: In the study part A—oral health (MMSE groups 1–5), the allocation was based on the order of participation in the study within the dementia groups 1–5 (even numbers—experimental group, uneven numbers—control group). In Part B—chewing function (MMSE groups 1–3), the allocation was based on the order of participation in the study within MMSE groups 1–3 but in reverse to Part A (even numbers—control group, uneven numbers—experimental group).

### 2.2. Study Objectives of the Planned Study

OrBiD—study part A—oral health.

The aims of the planned study part A were to identify differences in oral health between people with and without dementia as well as to show the influence of an intervention on oral health depending on the degree of dementia.

OrBiD—study part B—chewing function.

The aims of the planned study part B were to identify differences in chewing function between people with and without dementia as well as to show the influence of an intervention on chewing function depending on the degree of dementia.

The overall objective for both study parts was to find approaches and, if necessary, to develop concepts for better dental care and for the improvement of the chewing function for patients with dementia that will improve their quality of life.

**Table 1:** OrBiD study design including evaluation time-points, intervals between the evaluation time-points and the allocation to the experimental and control group in the study parts A—oral health and B—chewing function (Exp—experimental group, Con—control group) * See “Supplementary Material File S2 ([15,19,22,23,24,25,26,27,28,29,30,31,32,33,34,35,36,37])—Measuring instruments and evaluation methods” for an overview of measurements at individual evaluation time-points.

Evaluation Time-Point		MMSE Group 1 (MMSE 28–30) *n* = 24		MMSE Group 2 (MMSE 25–27) *n* = 24		MMSE Group 3 (MMSE 18–24) *n* = 24		MMSE Group 4 (MMSE 10–17) *n* = 24		MMSE Group 5 (MMSE ≤ 9) *n* = 24	
Baseline T0		Baseline evaluation of all subjects for study parts A and B (*n* = 120) *									
Assignment to Experimental group or Control group		Exp A Con B	Con A Exp B	Exp A Con B	Con A Exp B	Exp A Con B	Con A Exp B	Exp A	Con A	Exp A	Con A
		*n* = 12	*n* = 12	*n* = 12	*n* = 12	*n* = 12	*n* = 12	*n* = 12	*n* = 12	*n* = 12	*n* = 12
Inter vention starts directly after T0	Part A	Yes	No	Yes	No	Yes	No	Yes	No	Yes	No
	Part B	No	Yes	No	Yes	No	Yes	No Part B in MMSE groups 4 & 5			
T1 3 months after the start of the intervention		No evalu ation at T1/T2	Evalu ation *	No evalu ation at T1/T2	Evalu ation *	No evalu ation at T1/T2	Evalu ation *	No evaluation at T1/T2.			
T2 6 months after the start of the intervention											
Final 12 months after Baseline		Final evaluation of all subjects for study parts A and B (*n* = 120) *									

### 2.3. Working Hypotheses

The hypothesis is that the crossed study arm with people with dementia is possible and that the crossed study arms need fewer subjects than two separate studies. Specified objectives, separated by study parts A and B, and the resulting working hypotheses are presented in Table 2.

### 2.4. Measurements and Outcome Variables

In order to test the hypothesis of successful study design with the crossed study arms, assessment elements that are long-established were used in detail to avoid research bias. For the same reason, all clinical procedures and assessments were conducted by a single investigator. The measuring instruments and evaluation methods used in both parts of the study are described in the Supplementary Materials (Supplementary Material File S2, [15,19,22,23,24,25,26,27,28,29,30,31,32,33,34,35,36,37]).

### 2.5. Eligibility

The inclusion and exclusion criteria apply equally to all subjects, regardless of their participation in experimental group A or B.

Included were subjects aged 60 years and older regardless of their cognitive abilities, but with sufficient knowledge of German to be able to participate in an interview and to follow the intervention instructions during the evaluations and the intervention appointments. For subjects with acute dental processes (e.g., pain, abscesses, etc.) requiring emergency treatment, participation in the study started after successful emergency treatment. In addition, the subjects should not have any temporomandibular disorders (TMD). Furthermore, they should have had no or at most one dental hygiene session in the last 12 months before the start of the study participation. Substantial changes in dental status, including the fabrication of new prosthetic restorations during the observation period, lead to the exclusion of the data of this subject for the longitudinal analysis. It was a requirement for participation that at least one antagonistic contact (including prosthetic restoration) per jaw side and/or no non-occlusion in the posterior region was present. Likewise, there must have been at least one natural tooth in the upper or lower jaw that occludes with a natural tooth or a denture tooth (edentulousness in one jaw is possible). Subjects who were edentulous both in the upper and lower jaw are excluded from participation because the effects of the oral health intervention would not be detectable. Subjects with intolerance/allergy to toothpaste with high fluoride content (Duraphat^®^ toothpaste 5000 ppm, Colgate™) and/or phenylalanine were excluded.

Subjects with physical limitations in the upper body due to musculoskeletal or neuromuscular conditions, e.g., paralysis of the arms, arthritis, conditions after stroke with impairment of motor skills and facial nerve paralysis, etc., were excluded from the study-also if these changes only occur during the observation period. Furthermore, subjects with a congenital mental disability (e.g., Down’s syndrome, cerebral paresis) were excluded from participation.

Withdrawal of consent to participate in the study by the subjects or their legal representatives was possible at any time. Participation in the study would be terminated in case of undesirable side effects of the intervention. In study part A, this included the intolerance or development of allergies to the active ingredients contained in the highly fluoride-containing toothpaste (Duraphat^®^ toothpaste 5000 ppm, Colgate™). In study part B, undesirable side effects were muscular complaints or pain in the area of the jaw joints.

### 2.6. Interventions

Study part A—oral health.

All subjects of the experimental groups in study part A (MMSE groups 1 to 5) received a recommendation to intensify daily individual and professional oral and denture hygiene. This includes:

Recommendation to intensify the oral hygiene at home.

- Use of the highly fluoride-containing toothpaste (Duraphat^®^ 5000 ppm, Colgate™);
- At least twice a day instead of the toothpaste previously used;
- Use of the study toothpaste analogous to the use of conventional toothpaste (approx. pea-sized portion per toothbrush);
- Recommendation not to rinse, but only to spit out after brushing;
- Oral instruction of the subjects/relatives/ legal representatives/caregivers of the above-mentioned procedure by the investigator or a dental hygienist/prophylaxis assistant.

In addition, professional tooth and denture cleaning was also influenced.

- Increase in the professional prophylaxis frequency;
- Four appointments for professional oral and denture hygiene at intervals of three months within a year.

**Figure 1:**
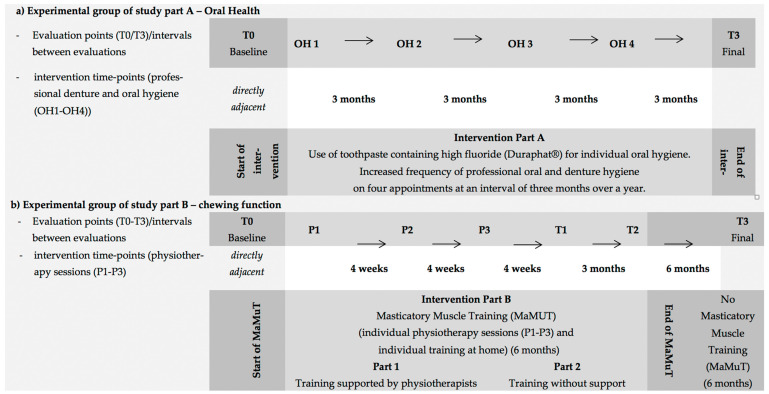
Timetable for evaluation points (T0/T3 for study part A—oral health, T0/T1/T2/T3 for study part B—chewing function) and intervention time-points (OH1–OH4 for study part A—oral health, P1–P3 for study part B—chewing function) for (**a**) experimental group of study part A—oral health and (**b**) experimental group of study part B—chewing function (OH—intervention part A, professional denture and oral hygiene; *p*—intervention part B—chewing function, physiotherapy sessions; MaMuT—masticatory muscle training).

This intervention was to be finally evaluated after 12 months after the baseline evaluation (Figure 1).

Study part B—chewing function.

In study part B, the intervention aims to train the masticatory muscles. Many frail people cannot chew well and are undernourished. The applied intervention, which aims to improve the chewing function in people with and without dementia, is completely innovative and, to our knowledge, has never been described in the literature before. It consists of three exercises and is carried out both with the support of a physiotherapist and through individual exercises at home for people without and with dementia (see also: Section 3.2. Novel methodology—Part B).

### 2.7. Securing Adherence to the Intervention

If subjects from both parts of the study obviously do not participate in the intervention or cannot participate due to their cognitive limitations, they were excluded from this part of the study for evaluation.

As a result of participating in the study, subjects in the experimental groups of study part A received four free professional oral hygiene sessions within 12 months. All subjects in all experimental and control groups receive free dental examinations (without X-ray examination) at the start and end of the study. This helped to ensure that participation in the study was highly compliant.

Study part A—oral health.

Subjects in the experimental group in part A of the study received the professional oral and denture hygiene prophylaxis appointments required for the intervention free of charge as well as the highly fluoride-containing toothpaste (Duraphat^®^ 5000 ppm) free of charge for the duration of the study.

Study part B—Chewing function.

Subjects of the experimental group in part B of the study received an expense allowance for each additional appointment at the ward or physiotherapist due to their participation in the intervention. Payment of the allowance was made after the completion of all scheduled evaluation and intervention appointments.

The following applies to both parts of the study: During the intervention, the experimental group in Part A—oral health was automatically the control group in Part B—chewing function, and vice versa. Both parts of the study were conducted parallel to each other.

Due to the difficulties in recruiting vulnerable subjects, in this case, people with dementia, in this pilot study, the subjects of the experimental group of one study part were simultaneously used as a control group in the other study part and vice versa. This resulted in less effort and better feasibility for recruiting the total required sample size per study part.

The authors assumed that there would be no profound influence of the respective intervention of the experimental group in one study part on the results of the control group in the other study part and vice versa. One reason for this is the study duration of one year. It is questionable to what extent better oral health, e.g., via tooth preservation (less tooth loss), can lead to improved chewing function in this short period of time. The effect of the Oral Health intervention can be expected in the short and medium-term, mainly for periodontal outcomes, such as a reduction in BOP or OHI index. It is unlikely that the Oral Health intervention leads to a reduced loss of teeth during the observation period and thus to a possibly improved ability to chew. Conversely, physiotherapeutic exercises were not expected to contribute to a measurable reduction in caries risk, e.g., via their possible effect on improved salivary secretion within the observation period. In order to observe such effects, a longer period than the intervention time targeted here was necessary. Second, it is unlikely that subjects in the experimental Oral Health group would simultaneously exercise their chewing ability independent of the study (e.g., through intensive gum chewing, which is not popular among the senior study population). Similarly, it is unlikely that subjects in the experimental chewing ability group would increase their frequency of professional dental cleanings with study entry. In addition, it cannot be ruled out that subjects in the Oral Health control group also randomly used the toothpaste used in the experimental group for daily oral hygiene at home.

The same inclusion and exclusion criteria were used to recruit all subjects. The initial situation of the control groups is therefore identical to that of the experimental groups.

The expected effect of the two interventions was unknown to all subjects. For this purpose, neither written nor verbal information was given to the subjects. Likewise, the implementation of the intervention as well as the measurement of the outcome variables was carried out without evaluating the measures or outcome variables to the subjects. This was to prevent subjects from drawing conclusions about the desired effect of the intervention to be achieved by the study, regardless of which control or experimental group they belonged to.

If abnormalities occur in the pilot study, this type of allocation to the control and experimental groups would have to be handled differently in a definitive study.

### 2.8. Sample Size Justification and Statistical Considerations

Since no literature related to the endpoints of the pilot study was found, power calculations were not performed. Therefore, from a statistical point of view, for the determination of the sample size, similarly designed studies and sample sizes used therein were used instead [38,39]. This results in a projected total sample size of 120 subjects for this pilot study. These 120 subjects were evenly distributed among the five MMSE groups (this corresponds to 24 subjects per MMSE group). Furthermore, the 24 subjects per MMSE group were equally allocated to the control or experimental group (i.e., per MMSE group *n* = 12 subjects in the test group and *n* = 12 subjects in the control group). This procedure should allow obtaining first assessments of both the influence of the mental status and the interventions in view of the outcome variables. Accordingly, subjects were recruited using stratified random sampling.

The statistical analyses correspond to the study design (cf. Table 1) and its nature as a pilot study. Both study parts A and B consist of a cross-sectional component at T0, comparing the effect of the mental status, and a longitudinal component, comparing the effects of the interventions. Besides descriptive and graphical tools, mixed linear models were tentatively used to account for the temporal dependency structure and to control for age and gender. If parametric model assumptions (e.g., normally distributed and homoscedastic residuals) are violated, robust non-parametric tests for the comparison between mental state and intervention effects were used.

Both Intention to Treat (ITT) and Per Protocol (PP) analyses were performed in order to account for potential deviations of the research protocol. Moreover, it was intended to replace subjects that changed their MMSE category during the time. It was possible that drop-outs would occur, especially for medical reasons or due to the death of the subject. Efforts were made to replace all subjects lost due to drop-outs with new recruitments. The remaining missing values were estimated by statistical means with a multivariate imputation algorithm [40]. 

An adjustment for effects resulting from medical procedures that may have occurred in the subjects during the observation period was not planned. Due to the vulnerability of the study population, it is certainly not excluded that, for example, the intake of new medications that influence salivary secretion could influence the outcome of the oral health part of the study. However, this represents realistic situations as they occur on a daily basis under non-study conditions. The aim of the study was also to show whether the planned interventions could be implemented in the everyday setting and lead to positive results. If other medical changes occurred, such as a stroke and the resulting reduction in motor function, these subjects would be excluded from further study participation and thus also from the longitudinal evaluation due to non-fulfillment of eligibility criteria. For the dental changes, there were several additional variables collected that would alert the authors to a severe change (e.g., a change in dental status resulting in a replacement of the prosthetic restoration, etc.). Subjects who showed irregularities in this regard during the observation period would be replaced by further recruitment.

### 2.9. Ethical Considerations

The study met the standards of the Declaration of Helsinki and of good clinical practice and was approved by the competent Cantonal Ethics Committee (CEC) of Zurich (KEK-ZH 2017-00363). All subjects or their legal representatives gave written informed consent.

## 3. Results

### 3.1. Novel Methodology

The OrBiD pilot study included two new methodological approaches. On the one hand, since the study focused on researching people with cognitive impairment and dementia in all degrees, a novel methodology for the recruitment process and in particular for the assignment of subjects to the experimental and control groups was developed (Novel methodology—part A: Novel recruitment process and unusual crossed allocation of subjects).

On the other hand, there is no study so far that attempts to positively influence bite force and chewing efficiency through the expertise of a physiotherapist with joint physiotherapy exercises and additional home exercises. For this reason, a complete physiotherapy program with targeted exercises to train strength and coordination of the masticatory muscles had to be newly developed. At the same time, the challenge was to ensure that this training could also be carried out with people with cognitive impairments and dementia, if necessary, in cooperation with their relatives or caregivers (Novel methodology—part B: Novel intervention to increase chewing function).

#### 3.1.1. Part A—Novel Recruitment Process and Unusual Crossed Allocation of Subjects to Support the Feasibility of Studies with Subjects with Dementia

Although OrBiD is a pilot study, a sufficient number of subjects is required to draw conclusions or develop clinical procedures from the results of the study. The authors attempted to meet these two challenges with the help of the novel methodology.

First, the recruitment process was adapted as the recruitment must also take place in different locations, depending on health, cognitive and organizational constraints.

**Figure 2:**
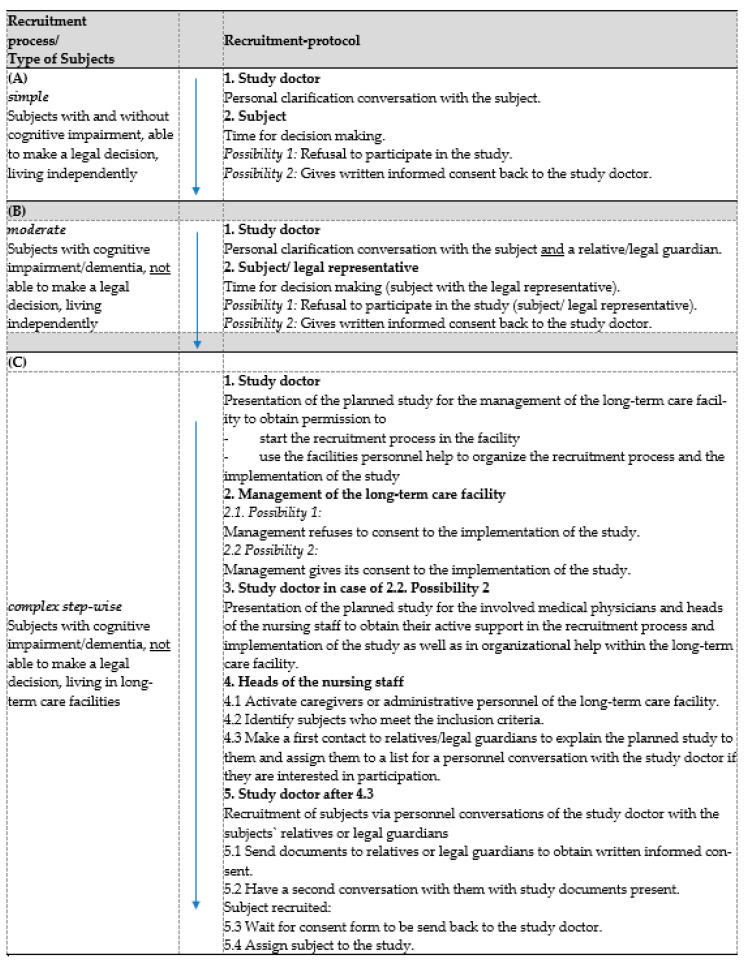
Recruitment process of study subjects depending on their cognitive condition, living situation and ability to obtain legal consent to study participation: simple, moderate or complex stepwise recruitment.

- For subjects with and without cognitive impairment, who were able to make a legal decision, and who were living independently, recruitment was based on the interaction between subject and study doctor (simple recruitment) (Figure 2A);
- For subjects with cognitive impairment or dementia, who were not able to make a legal decision but were living independently, the recruitment process was adapted. Here, relatives or close people were invited to take part in the decision process of the subject whether to participate in this study or not (moderate recruitment) (Figure 2B);
- For subjects with cognitive impairment and dementia, who were not able to make a legal decision, and who were living in long-term care facilities, the recruitment was carried out stepwise (complex stepwise recruitment) (Figure 2C).

In the second step, the allocation of recruited subjects was performed in an unconventional but necessary way: due to the complex and time-consuming recruitment process, especially in subjects with dementia, once subjects were recruited, they took crossed part in both study parts. During the trial, the subject in the experimental group in study part A—oral health, was also a subject in the control group in study part B—chewing function, and vice versa. Both parts of the study take place in parallel. One of the reasons for this is that the recruitment of people with dementia is significantly more difficult due to the involvement of legal representatives. Similarly, the authors rely on cooperation with other institutions, such as LTCF, to conduct the study. The time of the carers required for the study must be used efficiently; otherwise, the LTCF or the gerontological psychiatric institution cannot be expected to cooperate. The organizational, personnel, time and financial expenditure for all parties involved (study subjects and relatives, management of the care facility, participating physicians, investigators of the study) should be kept as low as possible.

#### 3.1.2. Part B—Novel Intervention to Increase Chewing Function

The intervention aims to train the masticatory muscles. As an intervention, subjects of the experimental group of study part B received a professional physiotherapeutic treatment to strengthen the masticatory muscles and to improve coordination during chewing (Masticatory Muscle Training (MaMuT)) by an experienced physiotherapist.

In three individual sessions of 30 min each, at intervals of four weeks (beginning 4 weeks after the baseline examination due to the coordination of the appointment), three exercises were performed with the subjects. Subjects or their relatives/caregivers were also instructed to carry out the MaMuT program independently at home. The subjects were instructed orally (if necessary, instruction of relatives/legal representatives/nursing staff in case of reduced cognition of the subjects). A control of the instructed exercises (independent demonstration of the exercises by the subject) was carried out directly after the instruction by the physiotherapist. The subjects and their relatives/caregivers were asked to repeat the exercises daily at home until the end of the training phase (four months after the last professional physiotherapy (P2) (Figure 1). In addition, all subjects received written and illustrated instructions with the exercises (Supplementary Material File S1) as well as the necessary material (hour-glass for monitoring the duration of the training per exercise and the duration of the break, chewing material for training bite force) for daily training at home. In each physiotherapeutic session, it was checked whether the subjects were able to perform the exercises independently and correctly. The subjects or, if appropriate, relatives/nursing staff were then corrected, re-instructed and motivated if necessary.

The MaMuT program consists of three physiotherapeutic exercises (Supplementary Material File S3):

- Exercise 1 was used for strength endurance training (aim: increasing bite force) and coordination (aim: increasing chewing efficiency). Therefore, the subjects were asked to chew a cube with 1 × 1 cm edge length (material: Permadyne Penta H, 3 M™ Espe™). Five training units of 30 s each and a 2 min break in between were performed with this exercise;
- Exercises 2 and 3 were two different isometric exercises to build up strength in the mastication muscles;
- Exercises 2 and 3 each: hold pressure for 5 s and then take a 5 s break, six repetitions, followed by a 2 min break; repeat each exercise three times).

The intervention started with a training phase supported by the physiotherapists (Figure 1b: intervention part 1—training supported by physiotherapists: three physiotherapeutic sessions within 2 months and daily individual home exercises). This was followed by a training phase without the support of the physiotherapist (Figure 1b—training without support: daily individual training at home only). After the first four weeks of individual daily training at home, the subjects were examined at T1 and three months later at T2 (end of the MaMuT program). After the T2 examination, the subjects were instructed not to perform the physiotherapeutic exercises anymore until the final examination (T3). Over a study period of 12 months, this resulted in a training phase (6 months) and a phase without training (6 months) (Figure 1).

In study part B, the effects of the intervention were assessed in the evaluation time-points T1 (3 months after the start of the intervention) and T2 (6 months after the start of the intervention)) and in a final evaluation (Final, 12 months after Baseline). (Table 1) Subjects with a higher degree of dementia (MMSE ≤ 17) in MMSE groups 4 and 5 were excluded from the intervention in part B of the study due to the severe cognitive impairment and the expected lack of ability to follow the intervention instructions. Subjects with mild dementia were included in the intervention to enable an assessment of the influence of dementia on chewing function. If effects were observed, it was then possible to develop specific treatment approaches for people with dementia that could be implemented at the onset of the disease.

### 3.2. Important Aspects of Feasibility

Important aspects of feasibility that may inform the design of a future definitive study need to be discussed.

The aim of the feasibility assessment of the planned pilot study was to review and evaluate its feasibility. For this purpose, the organizational effort, the required personnel, structural and financial resources, the required number of subjects and the type of study design should be investigated.

The following recommendations for a feasible study design for people with dementia can be made:

- Honestly state whether study hypotheses are at all testable for people with dementia;
- Study design with as few participants (subject, caregiver, relatives, study assistance, etc.) as possible so that the attention of the person with dementia is not wasted or they are not distracted;
- Reduced complexity of the study design by reducing the number of the groups of people involved in the study;
- A conscious choice of the study setting with the involvement of experienced people in dealing with people with dementia in order to optimize the organizational effort;
- Concentration and thus the limitation of the number of variables to be investigated to the essential items in order to ensure a burden for the study participants that are adapted to the clinical picture;
- Using a cross-study design to minimize recruitment burden;
- Use of over-recruitment to avoid loss of data due to expected increased drop-out rates.

### 3.3. Organizational Effort and Recruitment of Subjects

The organizational effort for the study implementation was to be estimated as large with regard to recruitment. The procedure described in the section “Novel recruitment process and unusual allocation of subjects” should make it feasible. However, a basic prerequisite for this procedure is a good personal, i.e., trustful and professional contact to the contact persons in the participating institutions. Furthermore, it is necessary to have experienced personnel for the recruitment and evaluation process who are trained in dealing with people with dementia and their relatives/legal guardians.

The motivation of the involved parties (investigators, contact persons in the facilities, involved personnel for the interventions) was an essential part of this complex study to be implemented.

### 3.4. Study Design Implications

Conducting the study without a respective control group would reduce (a) the effort and (b) the risk for possible effects of the control on the experimental groups. Nevertheless, the described study design should be maintained in the pilot study, since in this way, many findings on two important areas (oral health and chewing function) could be obtained in a study population that is often discriminated against in scientific studies. The length of the study should also be reviewed in the pilot study to find out how long a study session can be for vulnerable subjects or if long sessions need to be divided into more study visits. If problems result from the current study design, this would have to be adapted in a definitive study.

### 3.5. Results of Recruitment Based on the Application of the Study Design

This paper does not contain research results of the interventions of the OrBiD study itself.

The results of this methodological paper demonstrate that with the presented recruitment strategy, the targeted number of subjects can be recruited in a time frame acceptable for clinical trials. The method of the crossed study design has proven itself. In order to reach the targeted number of 24 subjects per study group 1–5 (n_total_ = 120), a total of 18 subjects had to be re-recruited during the course of the study (drop-out rate 21.6 % in relation to 120 subjects). Of these, 10 drop-outs (55.6 %) were subjects who lived independently at home, and 8 drop-outs were subjects from cooperating long-term care facilities (44.4%). For subjects living independently, reasons for drop-out included the withdrawal of consent to participate after the first appointment (*n* = 1), death of the subject (*n* = 2), health problems or hospitalization (*n* = 3) and pain, problems or excessive effort in performing the masticatory muscle training (*n* = 4). For subjects from collaborating long-term care facilities, drop-out reasons included the death of the subject (*n* = 4), withdrawal of consent to participate by relatives without providing further reasons (*n* = 3) and the subject moving away unknown (*n* = 1).

A total of 182 potential subjects were approached to participate in the study by the time it was completed. Of these, 99 potential subjects were part of the patient clientele of the clinic responsible for the study, and an additional 83 potential subjects were preselected by the collaborating institutions based on inclusion and exclusion criteria and were requested for official recruitment through the investigator. Of the 99 potential subjects from the organizing clinic’s patient clientele, 55 agreed to participate in the study (recruitment response rate 54.5%). All potential subjects of cooperating institutions or their legal representatives agreed to participate in the study (response rate of recruitment 100%).

### 3.6. Results of the Implementation of the MaMuT Programm

Likewise, the new method used for training the masticatory muscles was successfully implemented in the study. Of 36 subjects in the experimental group of study part B—chewing function who received the intervention, four subjects dropped out of the study because they either reported pain in the temporomandibular joint or under a denture after performing the exercises (*n* = 2) or experienced gag reflex when using the material to be chewed (*n* = 1). Another subject dropped out because the relatives of the subject living at home found the effort required for transportation to physiotherapy and for daily exercises too high.

## 4. Discussion

This methodological manuscript is a study protocol and includes a detailed description of new methodological approaches to recruiting vulnerable subjects. What is new is (A) a detailed description of the recruitment process of people with and without dementia in the attempt to reduce the time and personnel required for the recruitment process while at the same time increasing the recruitment rate and minimizing the drop-out rate and increasing adherence to study participation, and (B) the development and description of a completely new approach to increase bite force and chewing efficiency in people with and without dementia with the help of physical therapy exercises (Masticatory Muscle Training (MaMuT)).

### 4.1. Study Design

The study has the following limitations in study design.

With the crossed study design described, an attempt was made to include subjects in two study parts simultaneously.

With the increase in frailty and the development of dementia, the number of subjects who are cared for by relatives/legal guardians increases. Medical decisions are no longer made independently by the patients alone, so information and consent to participate in the study must then be provided for two people with different perceptions and interests (patients and relative/legal guardian). It should also be noted that sometimes the legal guardian or relative has no indication of how the patient would have decided to participate in research earlier in life. It can also be assumed that the compliance for the intervention is also directly or indirectly lower for subjects with a lower MMSE value (higher effort for the caregivers, more difficulty in finding an appointment, communication via third parties makes it more difficult, etc.). This can have an aggravating effect on the recruitment of the subjects. The authors assume, however, that a decision to participate in the study can be positively influenced both by the subjects themselves and by their relatives/legal guardians by the possibility of expense reimbursement. In particular, the possible prospect of four free professional oral and denture hygiene sessions within 12 months (study part A—Oral Health) could have a positive effect on recruitment (Switzerland: dental services are generally financed by the patient themselves; if financial security is low, funding from government agencies and in medically justified exceptions by health insurances) [41].

Recruitment must also take place in different locations, depending on health, cognitive and organizational constraints. Independently living patients are likely to be recruited at the clinic. Home-dwelling patients who are frail and dependent on the outpatient support of relatives or nursing services due to limited independence are also likely to be recruited at the clinic. In contrast, patients with severe physical or cognitive limitations will be sought out in LTCF or in geronto-psychiatric clinics for study information and consent, the oral findings and interventions in their facilities. For the planned interventions, both physiotherapists and the dental team must be mobile, e.g., use of a mobile unit for professional denture and tooth cleaning. This results in a high organizational and logistical study effort, which also requires higher financial support due to the increased time required per study subject.

Another reason for the simultaneous use of a subject in the experimental group of one study arm and in the control group of the other study arm is the expected high drop-out rate, which has its origin in the study clients themselves (older people, people with dementia).

Due to the clientele of study subjects, it can be assumed that drop-outs would occur for health or ethical reasons or due to the death of the subjects. The study subjects who were lost due to drop-outs will be attempted to be replaced with new recruitments in order to keep the numbers per MMSE group constant.

The simple and time-efficient Mini–Mental State Examination (MMSE) [34] is considered a suitable instrument to assess cognitive impairment in medical practice. It can be used as a basic diagnostic tool for the preliminary quantification of cognitive deficits and as an assessment of their severity [42]. It has to be considered that there may be a bias since subjects with cognitive impairments may already know the questions of the MMSE. Therefore, the results may be influenced. Furthermore, by using the MMSE, it is not possible to differentiate between types of dementia; the sensitivity is questionable, especially in mild dementia.

The assignment of the MMSE groups on the basis of the MMSE value was based on the stages of the MMSE values described by Perneczky et al. [43]. Since the present study tests the effects of interventions that could be integrated into everyday clinical life, the authors decided to use a slightly modified classification of the degree of dementia based on the clinically significant therapeutic ability of the patients [24], which are a prerequisite for the feasibility of the study interventions.

The basis for the decision of the arbitrary thresholds was an expert panel, which gave a range in the MMSE of 16–20 as a threshold for impairment in everyday performances (i.e., execution of the daily oral and denture hygiene, therapeutic ability with professional oral prophylactic procedures, execution and application of physiotherapeutic exercises).

In MMSE groups 4 and 5 (MMSE ≤17), there is no intervention in study part B—chewing function based on expert opinion that results from the clinical experience of the authors, which shows that subjects with these MMSE values are no longer reliable or even able to follow instructions of a complex intervention in a precise manner. Additionally, due to the reduced cognitive functions, severe impairments in the implementation of motor tasks are to be expected, which makes it impossible to show a possible long-term effect of the intervention (physiotherapeutic exercises to increase chewing function).

All examinations of the study are conducted by a non-blinded investigator. A bias can therefore not be completely ruled out. Nevertheless, the authors assume that the advantages for the study (no calibration necessary, no prior kappa analysis necessary) outweigh the disadvantages of having only one investigator, e.g., the consistency of the investigations, respondents with cognitive impairments are more responsive to a constant person during all examinations and participate more effectively in the examination. Moreover, the subjects are equally informed about the purpose of the study as well as the interventions. Therefore, a bias is possible, at least for the MMSE groups with no dementia, mild cognitive impairment or mild dementia (MMSE 30–18).

The study has the following potential benefits.

A benefit of participation is that patients with mostly limited or difficult access to dental services may receive regular, although time-limited, dental check-ups as a result of participating in the study. Subjects in study part A—oral health also benefit from an increased frequency of dental hygiene visits. Subjects in study part B—chewing function could also benefit from the intervention if a benefit can be achieved. However, it can be assumed that all subjects will benefit from increased patient-centered daily activity and attention by the caregivers, which can make their everyday life more sustainable for individual needs.

Recruitment also within LTCFs has the advantage that subjects do not have to leave their usual environment. This also reduces stress and anxiety, which in turn can have a positive effect on the collection of data and facilitate the implementation of the study personnel.

### 4.2. Outcome Variables and Measurements

There are limitations in this study with regard to the outcome variables and the related measurements. The limitations of the MMSE were already described in the previous section. In the evaluation of the Barthel index, the risk of bias arises due to different data collection (fit subjects: self-disclosure; frail subjects: a combination of self-disclosure and external assessment; subjects in need of care: assessment by the nursing staff). The bias is lowest in the group of persons in need of care since a third-party assessment is carried out. The risk of presenting a better self-image is, therefore, highest in healthy subjects but probably has the least influence since they are fit, and generally, high scores in the Barthel Index can be expected. Although various associations between chewing function and dementia are described in the literature (e.g., [44,45,46,47]), the method of carrying out the measurements (e.g., mixing ability test [15,17]) and their reliability in people with dementia should be questioned.

Often the physiological and mental state of people in need of care changes very quickly. This might also have a negative effect on all planned measurements. Reduced adherence to therapy in terms of the interventions or to the evaluation should also be discussed. It can be assumed for subjects with dementia who need care that the conduction of the daily individual exercises for the study part B intervention might only be achievable with the support of the nursing staff. Moreover, for the additional appointments for the increase in the professional oral hygiene frequency as well as the increase in the daily oral hygiene, the support of carers and/or relatives is necessary for cognitively impaired subjects. It is possible that these subjects show less compliance than subjects without dementia due to a lower understanding of the interventions and the study evaluations.

## 5. Conclusions

With this crossover study protocol, profound new methodological approaches for recruiting people with dementia as study subjects were described and successfully tested for their implementation. Subjects were included in two study parts at the same time to reduce the effort in the recruitment process and the study-related burden on subjects with dementia. When planning studies in this design, it must be ensured that the content, the interventions or their possible results of the study arms do not influence each other. In addition, a new physiotherapy program to train the masticatory muscles of people with and without dementia was developed (MaMuT program), and its use was successfully tested.

## Figures and Tables

**Table 2:** Objectives and working hypotheses for the crossed arm study part A—oral health (main influencing parameter increase in oral and denture hygiene) and study part B—chewing function (main influencing parameter physiotherapeutic exercises to train the masticatory muscles).

Study Part	Objectives	Hypotheses
**Part A**	Identification of factors influencing oral health, the use of dental services, the level of dental care and the oral health-related quality of life as a result of dementia (cross-sectional study)	- The degree of dementia of a subject has no influence on his DMF/T index.
		- The tooth and denture status shows no dependence on oral functional capacity.
		- The tooth and denture status depends on the cognitive abilities (degree of dementia).
		- Subjects with a lower degree of dementia show a better oral health-related quality of life.
		- The degree of dementia has an influence on the need for treatment.
	Description of the development and changes in oral health-related parameters in subjects with dementia as a function of dental care and treatment (recall frequency) and over time (longitudinal study)	- The increase in the utilization of dental services (increase in the recall frequency) has an influence on oral health-related parameters of people with dementia.
**Part B**	Clarification of the question of whether physiotherapeutic exercises to strengthen the masticatory muscles have an influence on chewing efficiency and bite force (longitudinal study)	- Physiotherapeutic exercises to strengthen the masticatory muscles show an effect in terms of improving bite force but not chewing efficiency in all subjects.
	Description of differences in chewing efficiency and bite force as well as possible differences in the effectiveness of exercises to strengthen the chewing muscles of a subject depending on the cognitive state (cross-sectional and longitudinal study)	- The cognitive state of a subject has an influence on the chewing efficiency and bite force.
	Identification of dependent variables of chewing efficiency and bite force (cross-sectional and longitudinal)	- The tooth and denture status and the cognitive function have an influence on chewing efficiency and bite force. - There is a correlation between chewing function and influencing factors (e.g., tooth and denture status, number of teeth and supporting zones, Mini Nutritional Assessment (MNA), body mass index (BMI), etc.). - There is a correlation between handgrip strength and chewing function as a function of dementia.

## Data Availability

Not applicable.

## Supplementary Materials

The following are available online at https://www.mdpi.com/article/10.3390/ijerph19063700/s1, Supplementary Material File S1: Explanation of terms; Supplementary Material File S2: Instruments and Evaluation Methods; Supplementary Material File S3: MaMuT program.
