# Supplementary material for: The OrBiD (Oral Health, Bite Force and Dementia) Pilot Study: A Study Protocol for New Approaches to Masticatory Muscle Training and Efficient Recruitment for Longitudinal Studies in People with Dementia
Source: Int J Environ Res Public Health. 2022 Mar 20;19(6):3700. doi: 10.3390/ijerph19063700 (PMC8992135; doi:10.3390/ijerph19063700)
Supplement: Supplementary file 1 [file ijerph-19-03700-s001.zip › Supplementary Material File S1 - Explanation of terms.pdf]

## Supplementary Material File S1 - Explanation of terms

**Oral health** describes the integrity of hard (e.g. tooth, bone) and soft tissue (e.g. tongue, oral mucosa, palate) in the oral cavity despite possible exposure to bacteria and their metabolic products (e.g. development of caries or periodontitis). Oral health can be promoted and maintained by regular, good oral hygiene.

**Chewing function** can be described by means of subjective chewing ability, and objective chewing efficiency and bite force.

The *chewing ability* describes the subjective chewing ability of a person, which is determined by questionnaires. [5, 6]

The *chewing efficiency* (also: masticatory efficiency) refers to the objectively measurable chewing performance that can be reproducibly evaluated by tests. Various objective test procedures are available for evaluating the chewing efficiency, which measure the degree of comminution of a defined quantity of food within a certain time or with a defined number of chewing cycles (e.g. sieve method [7, 8], computer-aided particle analysis [9], degree of carrot comminution [10]). A test procedure that is primarily used in patients with reduced chewing function [11] is the color mixing ability test according to J. F. Prinz [12, 13]. Here, the degree of mixing of the chewing material is measured as a quantitative measure of chewing efficiency. Comparative studies show that the results correlate significantly with those of the sieve method [14]. Modified forms of the color mixing ability tests, the two-color mixing ability tests, were described by Schimmel et al. [15] and by van der Bilt et al. [16]. Tests on mixing ability are objective measurements of chewing performance. [15-18] With the help of the two-color mixing test according to Schimmel, an objective assessment of the chewing function is possible. Both the visual analysis and the use of special software enable differentiation of chewing efficiency. [19] Hayakawa et al. also describe the evaluation of chewing performance using color-changing chewing gum [20] as a valid and reliable method [21].

The *bite force* (also: occlusal force, (maximum) occlusal force) provides information about the physiologically possible force of the test person to comminute a chewing item, but does not provide any information about the efficiency of the process.
