# Supplementary material for: The OrBiD (Oral Health, Bite Force and Dementia) Pilot Study: A Study Protocol for New Approaches to Masticatory Muscle Training and Efficient Recruitment for Longitudinal Studies in People with Dementia
Source: Int J Environ Res Public Health. 2022 Mar 20;19(6):3700. doi: 10.3390/ijerph19063700 (PMC8992135; doi:10.3390/ijerph19063700)
Supplement: Supplementary file 1 [file ijerph-19-03700-s001.zip › Supplementary Material File S2 - instruments_evaluation methods.pdf]

Supplementary Material File S2 - Measuring instruments and evaluation methods

Study part A – Oral health

- *DMF/T index* [22]

The DMF/T index (D - decayed, M - missing, F - filled, T - teeth) is described as a measure of caries experience. [22] The World Health Organization (WHO) criteria were used for decayed and filled teeth. Early stages of caries as well as stages of caries that precede cavitation were excluded from being recorded as decayed teeth. Older people often do not know why they had lost a tooth many years ago. Therefore, it is difficult to differentiate whether a tooth was lost due to caries or periodontal disease, or trauma. Thus, the DMF/T index has not been used as an accurate measure of caries experience in gerodontology, but rather as an epidemiological description for elderly people. [22, 23] Therefore, missing teeth (MT) in this analysis include teeth missing due to caries, periodontitis or trauma. The DMF/T index can be related to 28 or 32 teeth.

- *Degree of restoration*

The degree of restoration (in percent, %) was calculated from components of the DMF/T Index as follows: (F / D+F) x 100.

- *Oral functional capacity (OFC)*

Oral functional capacity (OFC) (Table 3) is used to assess patients from the multifactorial perspective of a gerostomatological and/or in special care working dentist. OFC is an assessment tool which helps to assess patients with regard to their resilience capacity level (RCL) with the three parameters therapeutic capability, oral hygiene ability and self-responsibility. The levels of the parameter’s therapeutic capability and oral hygiene ability range from 1 - normal to 2 - slightly reduced, 3 - greatly reduced and 4 - none. Self-responsibility is recorded with the capacity levels normal, reduced and none. The highest value of one of the three parameters determines the patient's resilience capacity level (RCL). For therapeutic capability an RCL 1 (normal) means that all therapy options can be performed, while RCL 4 (none) means that therapy options are extremely limited or non-existent due to cognitive and/or physical frailty of the patient. Oral hygiene ability RCL depends mostly on the question whether the patient has the ability to perform oral and prosthetic hygiene independently or whether a third person partially or completely performs the oral and prosthetic hygiene of the patient. Self-responsibility describes if the patient acts on his/her own responsibility, independently and autonomously.

The evaluation itself is independent of factors such as age, dental status and financial situation. It considers a variety of aspects influencing the feasibility of providing treatment to a patient. Some typical aspects to be considered when assessing therapeutic capability include but are not limited to the risk of general medical incidents, drug interactions, transportability, and limitations of patient positioning on the chair, feasibility of diagnostic procedure, manual dexterity and ability to open the mouth for longer periods. Within the scope of oral hygiene ability factors such as visual acuity, handgrip strength, and need of help with oral hygiene are assessed. Self-responsibility includes aspects like visiting behaviour/dental service uptake, expression of will, who is the responsible person for decisions, etc. [24]

Table 3 Classification of the Oral functional capacity with resilience capacity level (RCL) and the three parameters therapeutic capability, oral hygiene ability and self-responsibility [24]

Resilience capacity level (RCL)	Therapeutic capability	Oral hygiene ability	Self-responsibility
RCL 1<br>Normal	Normal	Normal	Normal
RCL 2<br>Slightly reduced	Slightly reduced	Slightly reduced	Normal
RCL 3<br>Greatly reduced	Greatly reduced	Greatly reduced	Reduced
RCL 4<br>No resilence	None	None	None

- *Tooth and denture status*

The tooth and denture status are assessed separately for the upper and lower jaw. It is the result of the combination of the tooth status (categories: fully edentulous, partially edentulous, edentulous) and the prosthetic status (three categories: no denture available/no dentures used, removable denture, fixed denture). The tooth and denture status are recorded in five categories (1 - fully dentate, no dentures or fixed dentures, 2 - partially dentate, fixed dentures, 3 - partially dentate, removable denture, 4 - partially dentate, no dentures available, 5 - edentulous, removable dentures). The combination "edentulous, no dentures available" is not applicable because of the inclusion criteria.

- *Type of denture*

The type of dentures (categories: complete denture, model cast prosthesis, temporary denture/molded clamp, temporary denture, denture/precision attachment, telescopic denture, hybrid denture) is recorded separately for the upper and lower jaw.

- *Oral hygiene index Greene and Vermillion* [25]

The OHI was developed in 1960 by John C. Green and Jack R. Vermillion to evaluate and classify oral hygiene. It provides a simple and reliable method to quantify the oral hygiene of individuals or groups. The OHI consists of two components: Debris Index (DI) and Calculus index (CI). The OHI is calculated according to the following rules. 1.) Only permanent, fully erupted teeth are evaluated. 2.) Wisdom teeth or teeth in infraocclusion are not evaluated. 3.) The score is collected for both DI and CI, per sextant, separately for buccal and lingual tooth surfaces. The tooth surface most covered by plaque or sub/supragingival calculus in a sextant is scored. The OHI can achieve a score between 0 and 3 (0 = no debris/calculus, 1 - soft plaque/ supragingival calculus covering not more than 1/3 of the tooth surface, 2 - soft plaque covering more than 1/3 but not more than 2/3 of the tooth surface or supragingival calculus or small areas of subgingival calculus in the cervical area, or both, 3 - soft plaque/ supragingival calculus covering more than 2/3 of the tooth surface or a wide band of subgingival calculus in the cervical area. It is calculated according to the following formula:

$$DI = \frac{\text{(Sum of buccal scores in the upper and lower jaw) + (Sum of lingual score in the upper and lower jaw)}}{\text{Number of segments assessed}}$$

$$CI = \frac{\text{(Sum of buccal scores in the upper and lower jaw) + (Sum of lingual score in the upper and lower jaw)}}{\text{Number of segments assessed}}$$

OHI = DI + CI

The higher the OHI (possible range 0-12), the worse the oral hygiene. DI and CI can each take a range of 0-6.

- *Mucosal examination → refer to: Study part B – Chewing function*

- *Halitosis*

The degree of halitosis of a subject is determined subjectively olfactorically by the investigator. Four categories are used: 1 - no halitosis, 2 - halitosis detectable at a distance of 10cm (low severity), 3 - halitosis detectable at a distance of 30cm (medium severity), 4 - halitosis detectable at a distance of 1m (high severity).

An objective measurement with the help of a halitometer cannot be performed due to the study design (inclusion of subjects in LTCF etc.) because of the required logistics.

- *Saliva quantity/quality*

The evaluation of saliva quality and quantity is performed subjectively by the investigator (categories: saliva quantity - moist, partially dry, dry; saliva quality - liquid, slightly foamy, no saliva).

On the other hand, an objective saliva measurement is carried out by determining resting saliva and stimulated saliva. The measurement should ideally be carried out in the morning in a fasting state.

To collect the data, the subject is first asked to spit the saliva that accumulates in the oral cavity into a container. The collection is carried out for 5 minutes. The amount of saliva collected is then weighed and the amount in ml divided by 5 minutes. The result is the amount of saliva at rest in ml/min. The amount of saliva at rest is categorized as follows: >1ml/min - hypersalivation, 0.25-1ml/min - norm salivation, 0.1-0.25 ml/min hyposalivation, <0.1ml/min xerostomia.

To determine the stimulated saliva, the participant is asked to chew a piece of paraffin for 5 minutes and not to swallow the saliva that is produced but to collect it in a container. The amount of saliva obtained is then weighed and the amount determined in ml divided by 5 minutes. The result is the stimulated saliva quantity in ml/min. The stimulated saliva quantity is categorized as follows: >3.5ml/min - hypersalivation, 1.0 - 3.5ml/min - norm salivation, 0.5 - 1.l/min - hyposalivation, <0.5ml/min - xerostomia.

- *The Periodontal Screening Index (PSI)* [26]

The PSI is collected as the worst value per sextant (code 0 - healthy, code 1 - gingivitis without calculus/plaque and without defective restoration margins, code 2 - gingivitis with calculus and/or plaque and/or defective restoration margins, code 3 - moderate periodontitis, code 4 - severe periodontitis). A WHO periodontal probe is used.

- *Bleeding on probing (BOP)*

BOP (categorical variable: yes/no) indicates the presence of bleeding caused by gentle manipulation of tissue at the depth of the gingival sulcus or the gingiva-to-tooth interface. If at least one site in the maxilla or mandible where probing depths have been measured with a periodontal probe (see Periodontal Screening Index) is positive (rating "yes"), BOP is rated as positive presence/yes overall.

- *Tooth mobility*

Tooth mobility is divided into degrees (degree 1 = none; degree 2 = visible; degree 3 = already movable at lip and tongue pressure or along the tooth axis).

- *Denture hygiene*

Dental prosthesis hygiene is assessed visually, ohne Anfärben by the dentist (categories: no dental plaque, plaque on the outer or inner surface of the prosthesis, calculus on the outer or inner surface of the prosthesis).

- *Uptake of dental services*

The number of visits to a dentist/oral hygienist per year two years prior the participation in the study is recorded.

- *OHIP-G14* [27]

The assessment of the oral health-related quality of life is carried out with the German version of the Oral Health Impact Profile (OHIP). A translated and validated short version with 14 questions is available in German (OHIP-G14), which can be answered by the respondent using a five-part Likert scale (never, hardly, occasionally, often, very often). The lower the total score in the OHIP (possible values are between 0 and 196), the better the oral health-related quality of life of the subject.

Study part B – Chewing function

- *Bite force/Maximum occlusal force (MOF)*

The measurement of the bite force (in Newton, N) at maximum voluntary clenching is performed with the occlusal force meter GM 10® (Morita, Nagano Keiki, Higashimagome, Ohta-ku, Tokyo, Japan) according to the procedure described in the literature [28] (Figure 4). The measurement is performed in the region of the first molar by applying the maximum possible jaw closing force. If the measurement was not possible on the first molar, the measurement was performed at the area closest to the first molar (e.g. 2nd molar/1st premolar) where the maximum possible voluntary biting force could be applied. Dentures were inserted, if available. All measurements were performed with the subjects in an upright sitting, unsupported position. The bite force is measured three times for each side of the jaw. The maximum occlusal force (MOF) of all measurements is included in the analysis.

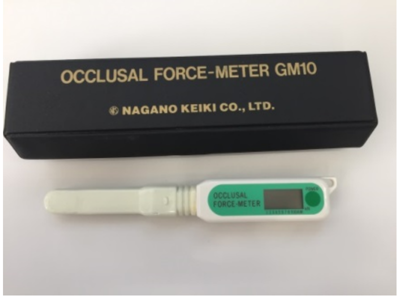

Figure 4 Occlusal Force Meter GM10™ for measuring the bite force in Newton

- *Chewing efficiency*

The chewing effciency was determined by means of the color-mixing ability test according to Schimmel et al. [15] Two chewing gums (Hue-check Gum®, Orophys GmbH, Muri b. Bern, Switzerland) were moistened and stuck together. The chewing gum was then placed on the tongue of the subjects. The subjects were instructed to chew 20 times (on the preferred chewing side). The gum was then removed from the mouth by the investigator. Immediately afterwards, the investigator performed the subjective visual assessment (5-step ordinal subjective assessment scale (SA) according to Schimmel et al. 2007 [15] (Figure 5): SA1 - chewing gum not mixed, impressions of cups or folded once; SA2 - large parts of chewing gum unmixed; SA3 - bolus slightly mixed, but bits of unmixed original color; SA4 - bolus well mixed, but color not uniform; SA5 - bolus perfectly mixed with uniform color). In the next step the resulting bolus was then pressed to a thickness of 1mm. The wafer was scanned at 300dpi on both sides with a flatbed scanner (HP Color LaserJet MFP M477fdn, Hewlett Packard, Palo Alto, California, USA) and opto-electronically analyzed with free software (ViewGum®, www.dhal.com). The program calculates the hue value as described by Schimmel et al. [19] as a ratio of the unmixed fraction of the chewing gum to the total pixel number in a fixed size template. The variance of hue (VOH) is a measure of the chewing efficiency. Inadequate mixing of colors as an expression of poor chewing efficiency will result in high VOH and vice versa. [15, 19]

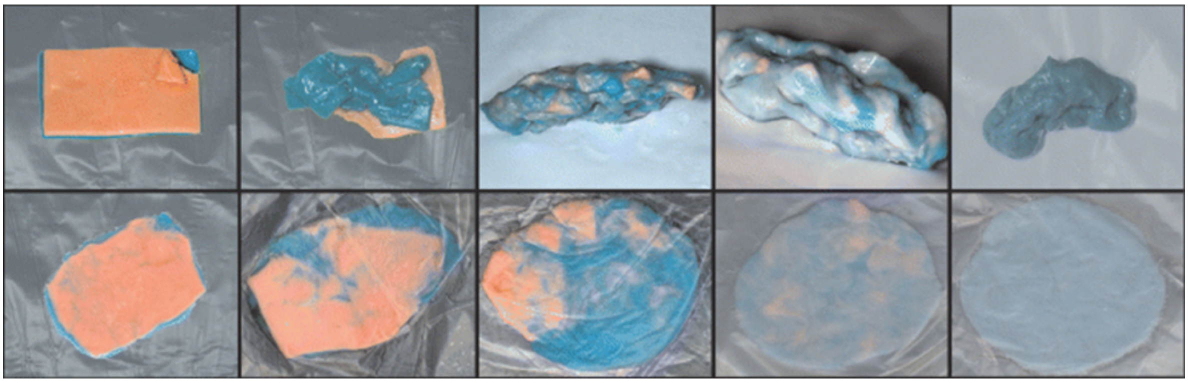

Figure 5 The five categories of the subjective assessment (SA) scale by Schimmel (2007) [15]. The upper line shows examples of the bolus in each class. The lower line shows the wafers which result from the boluses and are scanned for the opto-electronical analyzation.

- *Handgrip strength*

The handgrip strength measurement is carried out with the JAMAR® dynamometer, which is described as a valid measuring instrument. [29, 30] The patient sits upright on a chair, the measuring device is held vertically, with the upper arm suspended in a relaxed position and the forearm angled at an angle of 90 degrees. The wrist is slightly bent (about 30 degrees to the forearm). The examiner supports the measuring device from below and instructs the study participant to squeeze the grip handle as hard as possible. The measurement was performed three times for the dominant hand. The measurement is recorded three times for each side. The maximum value (in kilogram, kg) of both sides achieved by the participant, is included in the analysis.

- *Mucosal findings*

Furthermore, information is collected on mucosal diseases (whitish wipeable, whitish not wipeable, reddish changes, ulcerations, necrosis, pressure ulcera caused by dental prostheses).

- *Eichner index* [31]

With the Eichner index [31] the classification of the remaining dentition is performed. An assignment of fully, partially or edentulous patients is given. Decisive for the main groups A, B or C are the remaining supporting zones. A fully dentate patient with 14 teeth each in the upper and lower jaw has four supporting zones, two in the premolar and two in the molar region. Along the median sagittal plane, one premolar and one molar supporting zone is allocated to each half of the jaw. Existing dentures and third molars are not considered. The categories and subcategories for the Eichner grouping is shown in Table 4.

Table 4 Categories and subcategories of the Eichner index (Eichner 1955) [31]

**Categories/ subcategories**		**Description**
**A**		Intermaxillary contact in all occlusal supporting zones
	A1	Both jaws fully dentate, individual teeth decayed, but can be restored.
	A2	One jaw dentate, one jaw with interdental gaps
	A3	Both jaws with interdental gaps
**B**		Intermaxillary contact, not in all occlusal supporting zones
	B1	In three supporting zones
	B2	In two supporting zones
	B3	In one supporting zone
	B4	Occlusal contact outside the supporting zones
**C**		No intermaxillary contact
	C1	residual teeth in both jaws without antagonistic contact
	C2	One jaw edentulous, residual teeth in other jaw
	C3	Both jaws edentulous

- *Oral functional capacity (OFC) → refer to: study part A*

- *Tooth and denture status/type of denture→ refer to: study part A*

- *Number of supporting zones*

The number of support zones is assessed during the oral inspection. A complete dentition consists of four supporting zones, whereby the front teeth are not to be considered. A supporting zone consists of two opposing pairs of teeth (right and left premolars and molars), i.e. four teeth. There are four support zones resulting in 5 categories (0 none – 4 – four support zones).

- *Number of teeth*

The number of natural teeth with and without wisdom teeth in the entire dentition is counted. The number can therefore be related to 32 teeth (including the wisdom teeth) or to 28 teeth (not including the wisdom teeth).

- *Active/passive maximum mouth opening*

The active maximum mouth opening is the opening of the mouth that the subject is able to take on his own without any aids and without pain. The passive maximum mouth opening is the mouth opening supported by the investigator (in mm) up to a vertical limit position of the lower jaw. The investigator opens the mouth as wide as possible and places the thumb on the upper middle incisor and the index fingers on the lower one. In this position, moderate pressure is applied to move the lower jaw further to the border position. If a measurement is between two millimeters, the measurement is rounded off.

- *Overbite / Overjet*

The overbite indicates the vertical position of the front teeth. The distance from incisal edge to incisal edge is measured. The overjet indicates the positional relationship of the anterior teeth in the horizontal plane. The largest distance between the incisal edges of the central incisors is

measured at the upper and lower jaw. The overjet as well as the overbite is measured in millimeters (mm). An overjet of 0 mm indicates a frontal head bite, a negative overjet indicates progeny. An overjet of 0 mm indicates a sagittal step, a negative overjet indicates a frontal open bite. If a measurement lies between two-millimeter measurements, it is rounded off.

Study part A - Oral health <u>and</u> Study part B - Chewing function

- *Socio-demographic data*

Socio-demographic data such as age (in years), gender (male/female) and living conditions (living in LTCF /community-dwelling) are collected using a questionnaire.

- *Medical diagnoses and medications intake*

Information on the number and type (active substance classes) of the drugs prescribed to the subject as well as on general diseases is obtained by means of a questionnaire from the subject's medical doctor.

- *Mini Nutritional Assessment (MNA)* [32]

The MNA is a questionnaire which consists of a pre-assessment with six questions. A total of 14 points can be achieved in the pre-assessment. 12 points or more indicate a normal nutritional status, 11 points or less indicates the risk of malnutrition. The MNA main anamnesis contains 12 additional questions with a maximum score of 16 points. In the whole assessment (main anamnesis and pre-assessment) 30 points can be achieved. 17-23.5 points indicate being at risk for malnutrition. Less than 17 points indicate a poor nutritional status/malnutrition.

- *Body Mass Index (BMI)* [kg/m2]

The BMI is calculated by subjects' height and weight [kg/m^2]. [33]

- *Mini Mental State Examination*

The Mini Mental State Examination (MMSE) [34] which tests verbal and non-verbal episodic memory, orientation in time and place, visual-constructive abilities (maximum score 30) is used to assign the subjects to the evaluation groups. The MMSE is conducted by one dentist on all subjects who did not have a medical diagnosis of dementia and did not provide information on the MMSE value in their requested medical reports.

- *Denture quality* [35]

The assessment of denture quality was based on the following criteria: very good - no defects, no deviations from the ideal; good - acceptable quality, small deviations, corrections chairside possible; moderate - slight defects, correction by a dental technician necessary; poor - major defects, replacement required.

- *Dental treatment need*

The dental treatment need is an ordinal assessment of the necessity and urgency of dental treatment (objective or relative treatment need is either a) not necessary, b) immediate or acute or c) plannable). by the investigator. The assessment is based on the dental examination, the dental status, the oral hygiene findings and the tooth and denture status of the subjects. [24]

- *Barthel index* [36]

The Barthel Index is a method for the systematic recording of basic activities of daily living (ADL). In this process, ten different areas of activity are assessed. It is used to systematically record independence or the need for care and can reach a maximum score of 100 (no need of care). For frailty-affected seniors who are being cared for, the caregivers are asked to report their observation for the Barthel Index. The subjects who are not cared for are asked to fill out the Barthel Index for themselves, as no observation by others in daily life is possible here. If subjects are frail and are accompanied to the study dates by relatives/caregivers who can assess the current situation of the subject, the data for the Barthel Index are collected both with the subject themselves and with a caregiver in the same interview.

- *Geriatric Depression Scale (GDS)* [37]

The Geriatric Depression Scale according to Sheikh and Yesavage [37] contains 15 questions in the short form. GDS allows the early detection of possible depression as well as reproducible insights into the psychological state of ageing patients. GDS is often used for the initial assessment of a patient. It does not replace a specialist diagnosis. The cognitive situation should be clarified beforehand by means of the clock test or the Mini Mental Status in order to be able to judge whether the use of the Geriatric Depression Scale (GDS) is still necessary. It can reach a maximum number of 15 points.
