# Supplementary material for: The OrBiD (Oral Health, Bite Force and Dementia) Pilot Study: A Study Protocol for New Approaches to Masticatory Muscle Training and Efficient Recruitment for Longitudinal Studies in People with Dementia
Source: Int J Environ Res Public Health. 2022 Mar 20;19(6):3700. doi: 10.3390/ijerph19063700 (PMC8992135; doi:10.3390/ijerph19063700)
Supplement: Supplementary file 1 [file ijerph-19-03700-s001.zip › Supplementary Material File S3.pdf]

MaMuT program – Instructions for study participants

1

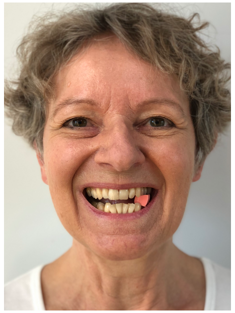

Strength endurance training and coordination

Please sit upright.
Put the silicone cube in your mouth and chew it without biting it.
Chew on both sides, please.
- 30 seconds chewing (approx. 25chewing cycles)
- 2 minutes break
Repeat the exercise including the breaks for 5 times.

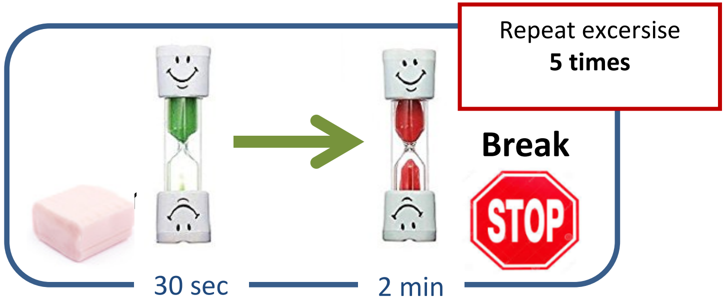

2

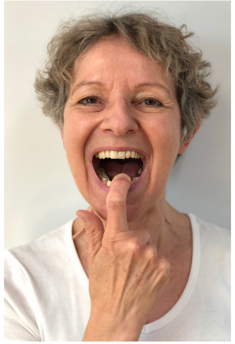

Building up strength in the lower jaw WITHOUT movements!

Please sit upright.
Open your mouth slightly and hang your hand with your finger on the lower teeth. Use your finger to apply pressure to the lower jaw downwards. At the same time you should try to close your mouth.
The lower jaw must not move.

3

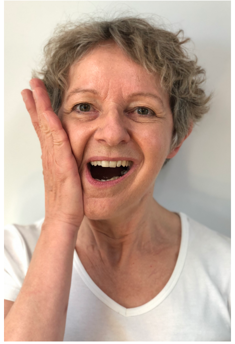

Building up strength in the cheek WITHOUT movement!

Please sit upright.
Place your right/left hand on the right or left side of the lower jaw. Press the hand against the lower jaw and at the same time press the lower jaw against the hand.
The lower jaw must not move.

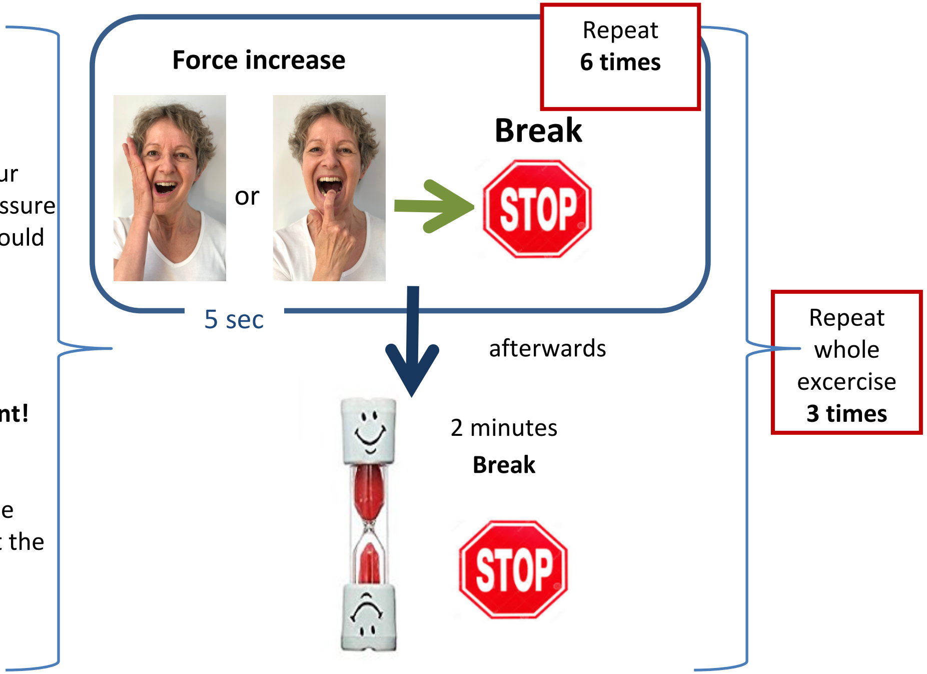

©Jockusch et al. 2020
